# Words matter: reclassifying chronic pruritus of unknown origin as neuroimmune pruritus

**DOI:** 10.1016/j.jdin.2026.04.002

**Published:** 2026-04-11

**Authors:** Hannah Verma, Nicholas Brownstone

**Affiliations:** Department of Dermatology, Icahn School of Medicine at Mount Sinai, New York, New York

**Keywords:** chronic pruritus of unknown origin, cytokines, itch, pruritus, type 2 inflammation

*To the Editor:* Chronic pruritus of unknown origin (CPUO) remains one of the most challenging diagnostic entities in dermatology. Traditionally defined as pruritus lasting longer than 6 weeks in the absence of identifiable systemic, dermatologic, neurologic, or psychiatric disease, CPUO has long been a diagnosis of exclusion. To address this, Kim et al^1^ proposed CPUO as uniform nomenclature to standardize diagnostic evaluation, prompting subsequent discussion regarding the classification, etiology, and heterogeneity of this entity. However, emerging advances in neurobiology and immunology increasingly support the concept that CPUO represents a neuroimmune disorder characterized by type 2 (Th2) inflammation and neuronal sensitization.

Contemporary models of itch no longer position pruritus as a purely symptomatic phenomenon secondary to cutaneous inflammation. Rather, itch is now recognized as a disease state characterized by alterations within peripheral and central neural circuits, sustained by immune-mediated signaling. Th2 cytokines, including interleukin (IL) 4, IL-13, and IL-31, directly activate and sensitize neurons.^2^ IL-4 and IL-13 signal through IL-4 receptor α and Janus kinase 1 pathways expressed on pruriceptive neurons, amplifying responsiveness to pruritogens.^2^ Advances in sensory neurobiology have identified sensory neuronal subsets [non-peptidergic (NP) 1–NP3 neurons] involved in itch. NP2 neurons expressing the Mas-related G protein-coupled receptor member A3 (MrgprA3) innervate the skin, reinforcing itch as a cutaneous sensory modality, while NP3 neurons express IL-31 receptor alpha, linking Th2 cytokine signaling to pruriceptive neuronal circuits.^2^ These findings demonstrate that immune mediators directly engage defined neuronal pathways in itch.

Histopathologic studies of skin biopsies from patients with chronic idiopathic pruritus demonstrate decreased Th1 cell signatures and increased Th2 polarization compared with controls, supporting a Th2-skewed inflammatory profile.^3^ Similarly, age-associated immune senescence, characterized by attenuation of Th1 immunity and relative predominance of Th2 inflammation, may predispose older adults to CPUO. This paradigm supports CPUO as a manifestation of neuroimmune dysregulation.

Therapeutic trial data reinforce this mechanistic framework. Conventional antipruritic approaches, particularly antihistamines, are frequently ineffective for patients with CPUO, consistent with evidence that many itch pathways are nonhistaminergic. In contrast, targeted modulation of Th2 and JAK-mediated signaling has demonstrated meaningful clinical benefit. A randomized clinical trial of the JAK1 inhibitor abrocitinib for chronic pruritus showed significant, clinically meaningful reductions in peak pruritus numerical rating scale scores and improvements in quality-of-life measures.^4^

Reclassification of CPUO as neuroimmune pruritus would have implications for research priorities, therapeutic development, and clinical care. The descriptor unknown origin implies absence of mechanism and may obscure the pathophysiology of this condition. In contrast, the term neuroimmune pruritus acknowledges defined immunologic and neuronal pathways and supports classifying CPUO as a Th2 inflammatory condition. As chronic itch is recognized as both a disease and a symptom, the terminology for CPUO warrants re-evaluation.^5^ The authors recognize that any change to disease nosology will likely require agreement through an Expert Delphi Consensus Panel. This more accurately reflects current evidence and supports the development of targeted therapies while improving diagnostic clarity.

## Conflicts of interest

None disclosed.
